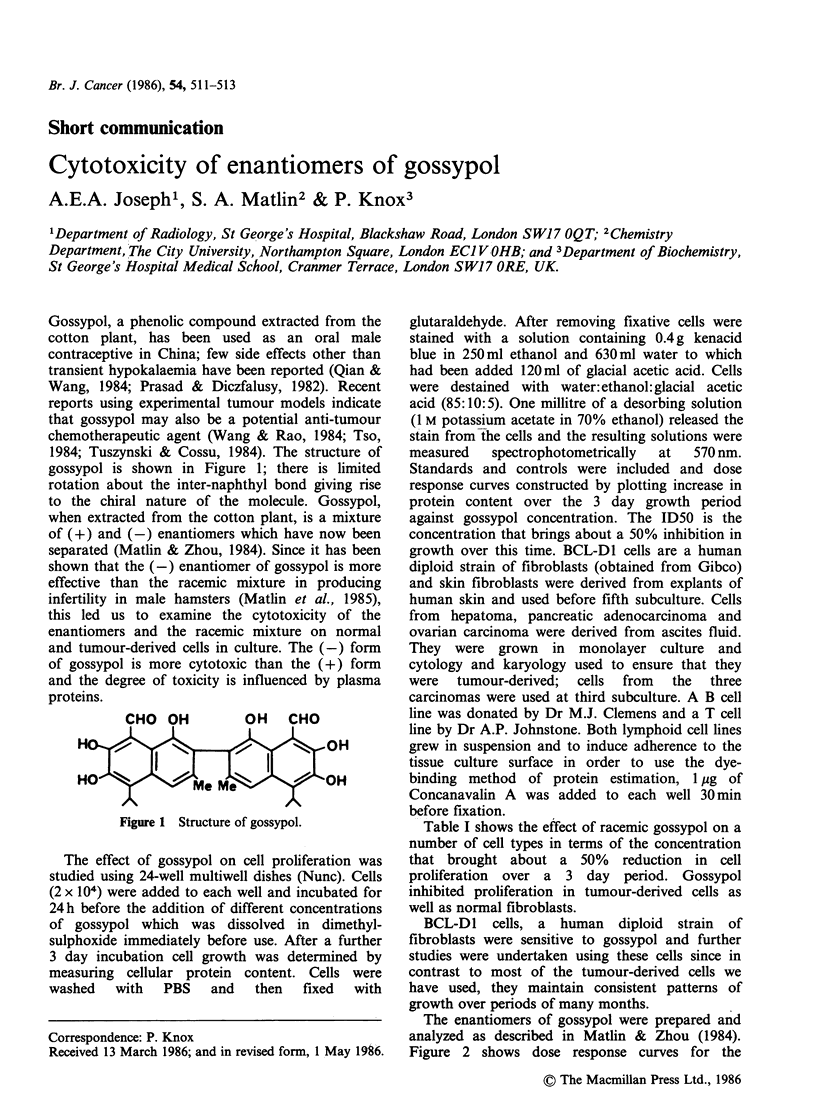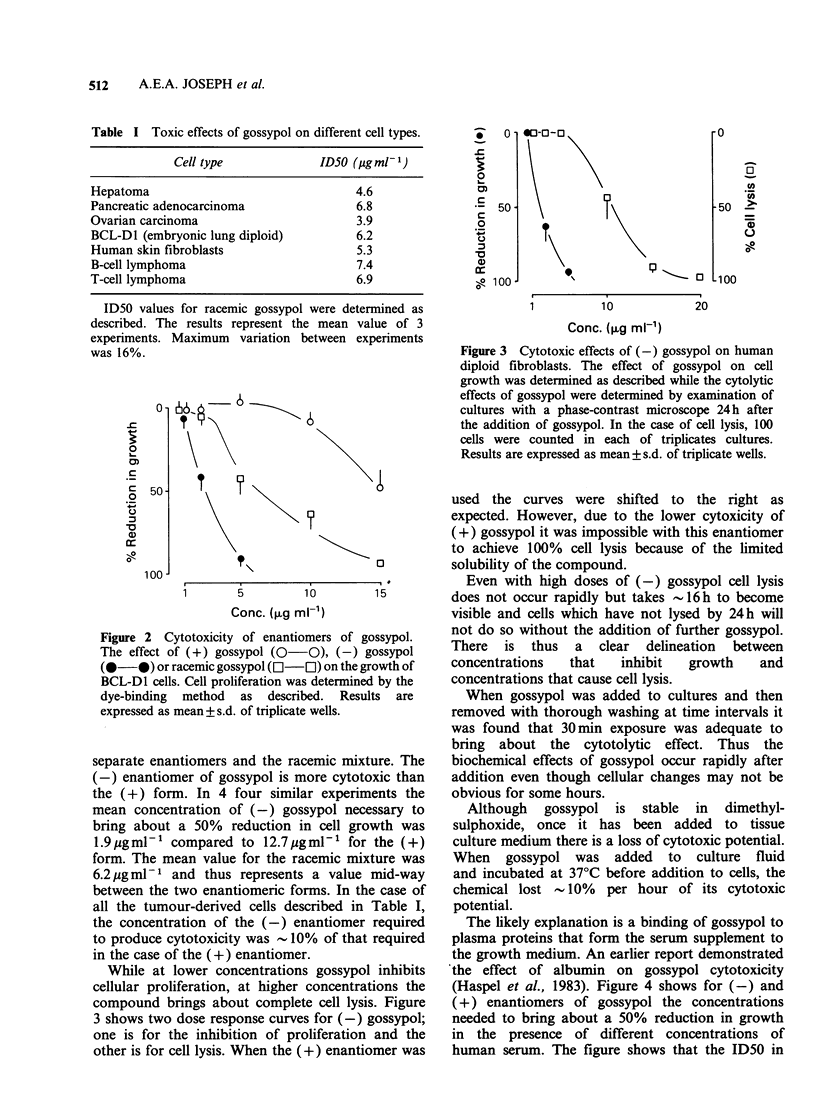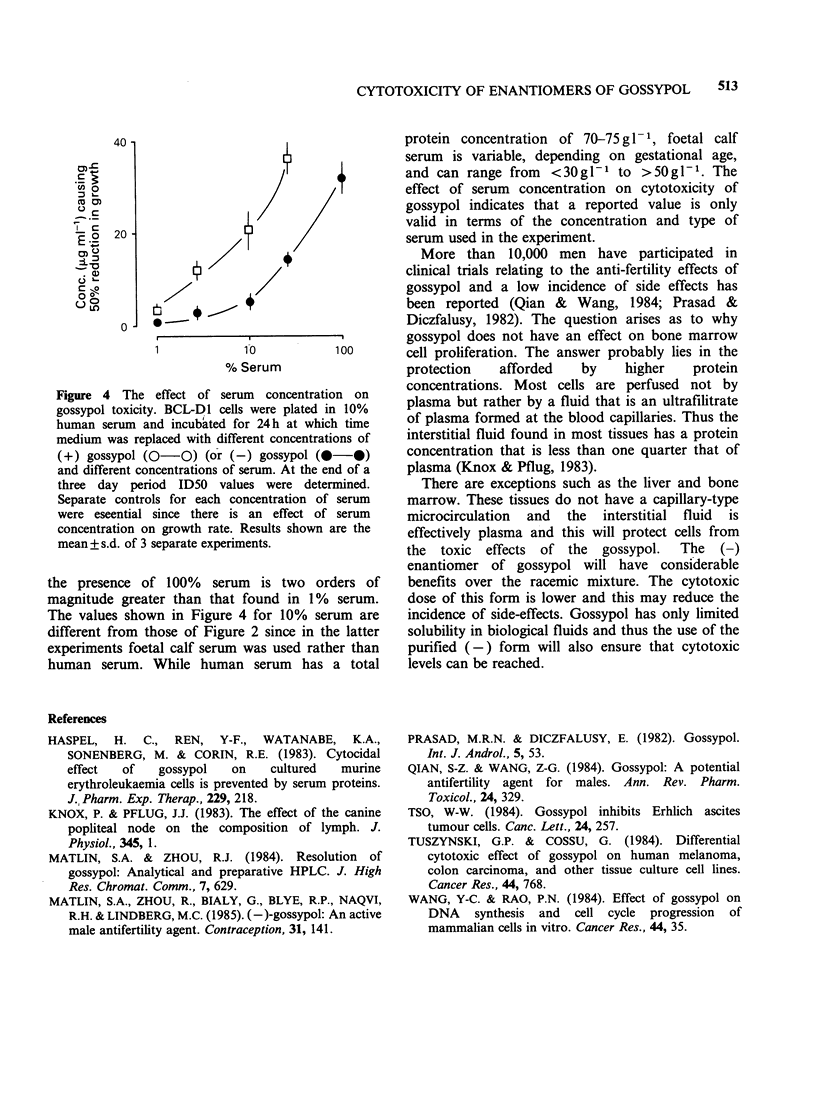# Cytotoxicity of enantiomers of gossypol.

**DOI:** 10.1038/bjc.1986.205

**Published:** 1986-09

**Authors:** A. E. Joseph, S. A. Matlin, P. Knox


*Br. J. Cancer* (1986), **54**, 511–513

**Short communication**

### Cytotoxicity of enantiomers of gossypol

A.E.A. Joseph[1], S. A. Matlin[2] & P. Knox[3]

[1]*Department of Radiology, St George's Hospital, Blackshaw Road, London SW17 0QT;* [2]*Chemistry Department, The City University, Northampton Square, London EC1V 0HB; and* [3]*Department of Biochemistry, St George's Hospital Medical School, Cranmer Terrace, London SW17 0RE, UK.*

Gossypol, a phenolic compound extracted from the cotton plant, has been used as an oral male contraceptive in China; few side effects other than transient hypokalaemia have been reported (Qian & Wang, 1984; Prasad & Diczfalusy, 1982). Recent reports using experimental tumour models indicate that gossypol may also be a potential anti-tumour chemotherapeutic agent (Wang & Rao, 1984; Tso, 1984; Tuszynski & Cossu, 1984). The structure of gossypol is shown in Figure 1; there is limited rotation about the inter-naphthyl bond giving rise to the chiral nature of the molecule. Gossypol, when extracted from the cotton plant, is a mixture of (+) and (−) enantiomers which have now been separated (Matlin & Zhou, 1984). Since it has been shown that the (−) enantiomer of gossypol is more effective than the racemic mixture in producing infertility in male hamsters (Matlin *et al.*, 1985), this led us to examine the cytotoxicity of the enantiomers and the racemic mixture on normal and tumour-derived cells in culture. The (−) form of gossypol is more cytotoxic than the (+) form and the degree of toxicity is influenced by plasma proteins.

**Figure 1** Structure of gossypol.

The effect of gossypol on cell proliferation was studied using 24-well multiwell dishes (Nunc). Cells ($2 \times 10^4$) were added to each well and incubated for 24 h before the addition of different concentrations of gossypol which was dissolved in dimethylsulphoxide immediately before use. After a further 3 day incubation cell growth was determined by measuring cellular protein content. Cells were washed with PBS and then fixed with glutaraldehyde. After removing fixative cells were stained with a solution containing 0.4 g kenacid blue in 250 ml ethanol and 630 ml water to which had been added 120 ml of glacial acetic acid. Cells were destained with water:ethanol:glacial acetic acid (85:10:5). One millitre of a desorbing solution (1 M potassium acetate in 70% ethanol) released the stain from the cells and the resulting solutions were measured spectrophotometrically at 570 nm. Standards and controls were included and dose response curves constructed by plotting increase in protein content over the 3 day growth period against gossypol concentration. The ID50 is the concentration that brings about a 50% inhibition in growth over this time. BCL-D1 cells are a human diploid strain of fibroblasts (obtained from Gibco) and skin fibroblasts were derived from explants of human skin and used before fifth subculture. Cells from hepatoma, pancreatic adenocarcinoma and ovarian carcinoma were derived from ascites fluid. They were grown in monolayer culture and cytology and karyology used to ensure that they were tumour-derived; cells from the three carcinomas were used at third subculture. A B cell line was donated by Dr M.J. Clemens and a T cell line by Dr A.P. Johnstone. Both lymphoid cell lines grew in suspension and to induce adherence to the tissue culture surface in order to use the dye-binding method of protein estimation, 1 μg of Concanavalin A was added to each well 30 min before fixation.

Table I shows the effect of racemic gossypol on a number of cell types in terms of the concentration that brought about a 50% reduction in cell proliferation over a 3 day period. Gossypol inhibited proliferation in tumour-derived cells as well as normal fibroblasts.

BCL-D1 cells, a human diploid strain of fibroblasts were sensitive to gossypol and further studies were undertaken using these cells since in contrast to most of the tumour-derived cells we have used, they maintain consistent patterns of growth over periods of many months.

The enantiomers of gossypol were prepared and analyzed as described in Matlin & Zhou (1984). Figure 2 shows dose response curves for the

Correspondence: P. Knox
Received 13 March 1986; and in revised form, 1 May 1986.

© The Macmillan Press Ltd., 1986

**Table I** Toxic effects of gossypol on different cell types.

| *Cell type* | *ID50 ($\mu g\, ml^{-1}$)* |
|---|---|
| Hepatoma | 4.6 |
| Pancreatic adenocarcinoma | 6.8 |
| Ovarian carcinoma | 3.9 |
| BCL-D1 (embryonic lung diploid) | 6.2 |
| Human skin fibroblasts | 5.3 |
| B-cell lymphoma | 7.4 |
| T-cell lymphoma | 6.9 |

ID50 values for racemic gossypol were determined as described. The results represent the mean value of 3 experiments. Maximum variation between experiments was 16%.

**Figure 2** Cytotoxicity of enantiomers of gossypol. The effect of (+) gossypol (○——○), (−) gossypol (●——●) or racemic gossypol (□——□) on the growth of BCL-D1 cells. Cell proliferation was determined by the dye-binding method as described. Results are expressed as mean ± s.d. of triplicate wells.

separate enantiomers and the racemic mixture. The (−) enantiomer of gossypol is more cytotoxic than the (+) form. In 4 four similar experiments the mean concentration of (−) gossypol necessary to bring about a 50% reduction in cell growth was $1.9\,\mu g\, ml^{-1}$ compared to $12.7\,\mu g\, ml^{-1}$ for the (+) form. The mean value for the racemic mixture was $6.2\,\mu g\, ml^{-1}$ and thus represents a value mid-way between the two enantiomeric forms. In the case of all the tumour-derived cells described in Table I, the concentration of the (−) enantiomer required to produce cytotoxicity was ~10% of that required in the case of the (+) enantiomer.

While at lower concentrations gossypol inhibits cellular proliferation, at higher concentrations the compound brings about complete cell lysis. Figure 3 shows two dose response curves for (−) gossypol; one is for the inhibition of proliferation and the other is for cell lysis. When the (+) enantiomer was

**Figure 3** Cytotoxic effects of (−) gossypol on human diploid fibroblasts. The effect of gossypol on cell growth was determined as described while the cytolytic effects of gossypol were determined by examination of cultures with a phase-contrast microscope 24 h after the addition of gossypol. In the case of cell lysis, 100 cells were counted in each of triplicates cultures. Results are expressed as mean ± s.d. of triplicate wells.

used the curves were shifted to the right as expected. However, due to the lower cytoxicity of (+) gossypol it was impossible with this enantiomer to achieve 100% cell lysis because of the limited solubility of the compound.

Even with high doses of (−) gossypol cell lysis does not occur rapidly but takes ~16 h to become visible and cells which have not lysed by 24 h will not do so without the addition of further gossypol. There is thus a clear delineation between concentrations that inhibit growth and concentrations that cause cell lysis.

When gossypol was added to cultures and then removed with thorough washing at time intervals it was found that 30 min exposure was adequate to bring about the cytotolytic effect. Thus the biochemical effects of gossypol occur rapidly after addition even though cellular changes may not be obvious for some hours.

Although gossypol is stable in dimethylsulphoxide, once it has been added to tissue culture medium there is a loss of cytotoxic potential. When gossypol was added to culture fluid and incubated at 37°C before addition to cells, the chemical lost ~10% per hour of its cytotoxic potential.

The likely explanation is a binding of gossypol to plasma proteins that form the serum supplement to the growth medium. An earlier report demonstrated the effect of albumin on gossypol cytotoxicity (Haspel *et al.*, 1983). Figure 4 shows for (−) and (+) enantiomers of gossypol the concentrations needed to bring about a 50% reduction in growth in the presence of different concentrations of human serum. The figure shows that the ID50 in

**Figure 4** The effect of serum concentration on gossypol toxicity. BCL-D1 cells were plated in 10% human serum and incubated for 24h at which time medium was replaced with different concentrations of (+) gossypol (○—○) (or (−) gossypol (●—●) and different concentrations of serum. At the end of a three day period ID50 values were determined. Separate controls for each concentration of serum were eseential since there is an effect of serum concentration on growth rate. Results shown are the mean ± s.d. of 3 separate experiments.

the presence of 100% serum is two orders of magnitude greater than that found in 1% serum. The values shown in Figure 4 for 10% serum are different from those of Figure 2 since in the latter experiments foetal calf serum was used rather than human serum. While human serum has a total protein concentration of 70–75 $g\,l^{-1}$, foetal calf serum is variable, depending on gestational age, and can range from $<30\,g\,l^{-1}$ to $>50\,g\,l^{-1}$. The effect of serum concentration on cytotoxicity of gossypol indicates that a reported value is only valid in terms of the concentration and type of serum used in the experiment.

More than 10,000 men have participated in clinical trials relating to the anti-fertility effects of gossypol and a low incidence of side effects has been reported (Qian & Wang, 1984; Prasad & Diczfalusy, 1982). The question arises as to why gossypol does not have an effect on bone marrow cell proliferation. The answer probably lies in the protection afforded by higher protein concentrations. Most cells are perfused not by plasma but rather by a fluid that is an ultrafilitrate of plasma formed at the blood capillaries. Thus the interstitial fluid found in most tissues has a protein concentration that is less than one quarter that of plasma (Knox & Pflug, 1983).

There are exceptions such as the liver and bone marrow. These tissues do not have a capillary-type microcirculation and the interstitial fluid is effectively plasma and this will protect cells from the toxic effects of the gossypol. The (−) enantiomer of gossypol will have considerable benefits over the racemic mixture. The cytotoxic dose of this form is lower and this may reduce the incidence of side-effects. Gossypol has only limited solubility in biological fluids and thus the use of the purified (−) form will also ensure that cytotoxic levels can be reached.

### References

HASPEL, H. C., REN, Y-F., WATANABE, K.A., SONENBERG, M. & CORIN, R.E. (1983). Cytocidal effect of gossypol on cultured murine erythroleukaemia cells is prevented by serum proteins. *J. Pharm. Exp. Therap.*, **229**, 218.

KNOX, P. & PFLUG, J.J. (1983). The effect of the canine popliteal node on the composition of lymph. *J. Physiol.*, **345**, 1.

MATLIN, S.A. & ZHOU, R.J. (1984). Resolution of gossypol: Analytical and preparative HPLC. *J. High Res. Chromat. Comm.*, **7**, 629.

MATLIN, S.A., ZHOU, R., BIALY, G., BLYE, R.P., NAQVI, R.H. & LINDBERG, M.C. (1985). (−)-gossypol: An active male antifertility agent. *Contraception*, **31**, 141.

PRASAD, M.R.N. & DICZFALUSY, E. (1982). Gossypol. *Int. J. Androl.*, **5**, 53.

QIAN, S-Z. & WANG, Z-G. (1984). Gossypol: A potential antifertility agent for males. *Ann. Rev. Pharm. Toxicol.*, **24**, 329.

TSO, W-W. (1984). Gossypol inhibits Erhlich ascites tumour cells. *Canc. Lett.*, **24**, 257.

TUSZYNSKI, G.P. & COSSU, G. (1984). Differential cytotoxic effect of gossypol on human melanoma, colon carcinoma, and other tissue culture cell lines. *Cancer Res.*, **44**, 768.

WANG, Y-C. & RAO, P.N. (1984). Effect of gossypol on DNA synthesis and cell cycle progression of mammalian cells in vitro. *Cancer Res.*, **44**, 35.